# Three novel aquatic species of *Distoseptispora* (Distoseptisporaceae, Distoseptisporales) from the Philippines, with a new geographic record for *D. septata*

**DOI:** 10.3897/mycokeys.137.198252

**Published:** 2026-07-21

**Authors:** Jan Felnesh Exe Bagacay, Gerlie Mae Candole, Jasmine C. Velo, Fred Ric Sara, Majoerly Augustine Siccio, Lilcah Angelique Opiña, E.B. Gareth Jones, Mark S. Calabon

**Affiliations:** 1 Division of Biological Sciences, College of Arts and Sciences, University of the Philippines Visayas, Miagao, Iloilo 5023, Philippines Division of Biological Sciences, College of Arts and Sciences, University of the Philippines Visayas Iloilo Philippines; 2 Graduate School, University of the Philippines Visayas, Iloilo City, Iloilo 5000, Philippines Graduate School, University of the Philippines Visayas Iloilo Philippines; 3 Philippine Genome Center Visayas, University of the Philippines Visayas, Miagao, Iloilo 5023, Philippines Philippine Genome Center Visayas, University of the Philippines Visayas Miagao Philippines; 4 IUCN SSC Aquatic Fungi Specialist Group, IUCN Species Survival Commission, Rue Mauverney 28, 1196 Gland, Switzerland IUCN SSC Aquatic Fungi Specialist Group, IUCN Species Survival Commission Gland Switzerland; 5 Nantgaredig, 33B St. Edwards Road, Southsea, Hants., PO5 3DH, UK Nantgaredig Southsea United Kingdom

**Keywords:** Aquatic fungi, fungal systematics, life below water, Philippine biodiversity, SDG 14, three new species

## Abstract

Surveys of lignicolous aquatic fungi conducted in the Philippines, yielded four taxa of *Distoseptispora*. Multi-locus phylogenetic analyses, based on a concatenated dataset of ITS, LSU, RPB2 and TEF1-α sequences, together with detailed morphological examinations, confirmed the novelty and phylogenetic placement of *Distoseptispora antiqueana*, *D. iloiloensis* and *D. panayensis* as distinct species within the genus. In addition, the strain UPVMICC 25–0100 clustered with the ex-type strain of *D. septata* (GZCC 22–0078) with strong statistical support (100% MLBS, 1.00 BYPP). Phylogenetic inference further supported taxonomic revisions within the genus, resulting in the synonymisation of *D. thailandica* under *D. phangngaensis* and *D. mengsongensis* under *D. fasciculata*. The present study expands the known diversity of *Distoseptispora* in tropical aquatic habitats and provides updated phylogenetic insights and morphological descriptions supporting species delimitation and reclassification within the genus.

## Introduction

Aquatic fungi colonise a wide array of natural and artificial substrates across diverse aquatic systems, including rivers, streams, lakes, deep-sea and sub-seafloor environments, hydrothermal vents, coastal and oceanic regions, polar habitats, aquifers, water cooling water, wastewater systems and even extreme environments, such as nuclear reactors, where they contribute significantly to ecosystem functioning and aquatic food web stability (Gulis et al. 2008; Wurzbacher et al. 2011; Wurzbacher et al. 2014; Grossart et al. 2019). Amongst these, lignicolous aquatic fungi — those inhabiting submerged woody substrates — play pivotal roles in carbon and nutrient cycling by decomposing lignocellulosic materials, thereby facilitating wood softening and nutrient release (Björdal 2012; Hyde et al. 2016a; Calabon et al. 2023a; Shen et al. 2025). Taxonomically, most aquatic fungi are classified within Dothideomycetes and Sordariomycetes, with current estimates indicating approximately 2,319 marine fungal species and about 3,870 freshwater species (Jones et al. 2019; Calabon et al. 2022; Calabon et al. 2023b). Recent studies have increasingly documented aquatic fungal diversity, particularly from Asian regions such as China and Thailand, with numerous reports of novel taxa and new records (Hyde et al. 2019; Luo et al. 2019; Dayarathne et al. 2020; Hyde et al. 2020; Dong et al. 2020; Dong et al. 2021; Bao et al. 2023), whereas in the Philippines, comprehensive taxonomic assessments and baseline data on aquatic fungal diversity remain relatively scarce (Jones et al. 1988).

One of the more species-rich, yet monotypic orders within Sordariomycetes is Distoseptisporales, placed in the subclass Diaporthomycetidae. This order was established by Luo et al. (2019) to accommodate the family Distoseptisporaceae and the genus *Aquapteridospora* (treated as genera *incertae sedis* within Distoseptisporales), based on morphological characteristics and combined phylogenetic analyses of LSU, SSU, RPB2 and TEF1-α sequence data. Su et al. (2016) established Distoseptisporaceae to accommodate the genus *Distoseptispora* typified by *D. fluminicola*. It is commonly associated with woody substrates in both freshwater and terrestrial habitats, where it plays an important role in lignocellulose degradation (Hyde et al. 2020). The majority of the genera are characterised, based on asexual morphs, largely relying on conidial morphology, including septation type (either distoseptate or euseptate), conidial size, shape and pigmentation for species identification (Su et al. 2016; Luo et al. 2019). The genus is predominantly reported from Asia, particularly China and Thailand, with relatively few records from other regions (Luo et al. 2019; Phukhamsakda et al. 2020; Zhai et al. 2022; Liao et al. 2025; He et al. 2026; Tian et al. 2026). Subsequent studies, including those on *D. guttulata* and *D. suoluoensis* (Yang et al. 2018), have refined the definition of the genus, while the discovery of distinct species, such as *D. palmarum* distinguished by the presence of polyblastic conidiogenous cells (Hyde et al. 2019), *D. appendiculata* characterised by a gelatinous, hyaline appendage sheath (Luo et al. 2019) and *D. hydei* which exhibits longer obclavate conidia with a higher number of septa (Monkai et al. 2020), has broadened its taxonomic limits by expanding the range of recognised morphological characteristics within the genus.

This study represents the first documented occurrence of *Distoseptispora* in Philippine aquatic habitats. Three novel species of *Distoseptispora* are introduced, including *D. antiqueana*, *D. iloiloensis* and *D. panayensis*, based on detailed morphological characterisation and multi-locus phylogenetic analyses. In addition, a new habitat and geographic record is reported for *D. septata*, extending its known ecological and distributional range. These findings contribute to the growing understanding of aquatic fungal diversity in Southeast Asia and emphasise the Philippines, particularly the Visayas Region, as an important, but understudied centre of fungal biodiversity.

## Materials and methods

### Sample collection, morphological observation and isolation

Submerged decaying wood was collected from aquatic habitats in Panay and Nogas Island, Philippines. Samples were incubated in moist chambers at room temperature for two weeks to induce sporulation. Fungal fruiting bodies were examined directly on the substrate using a Kuaiqu Simul-Focal trinocular dissecting microscope (Shenzhen Kuaiqu Electronic Co., Ltd., Shenzhen City, Guangdong Province, China). Micromorphological characters were observed and photographed using an Olympus BX53 (Olympus Corporation, Tokyo, Japan) compound microscope, equipped with a digital camera. Measurements were taken from at least 20 structures per taxon where possible. Photographic plates were prepared using Adobe Photoshop 2026 (Adobe 2026). Single-spore isolation was performed following Senanayake et al. (2020). Germinated spores were transferred to potato dextrose agar (PDA) and incubated at 25 °C. Colony characteristics (diameter, colour, texture, margin and pigmentation) were recorded after 7–14 days. Voucher specimens were deposited in the University of the Philippines Visayas Mycological Herbarium (UPVMI). Living cultures are preserved in the University of the Philippines Visayas Microbial Culture Collection (UPVMICC).

### DNA extraction, PCR amplification and sequencing

Genomic DNA was extracted from fresh mycelia grown on PDA using the DNeasy Blood and Tissue Kit (Qiagen, Germany) following the manufacturer’s protocol. Four loci were amplified: the internal transcribed spacer region (ITS), the large subunit of ribosomal RNA (LSU), partial second largest subunit of RNA polymerase II (RPB2) and the TEF1-α. Primer pairs ITS5/ITS4 (White et al. 1990) were used for ITS, LR0R/LR5 (Vilgalys and Hester 1990) for LSU, RPB2–5F2/7cR for RPB2 (Liu et al. 1999) and EF1–983F/EF1–2218R for TEF1-α (Tanaka et al. 2023). PCR reactions were carried out in 25 μl volumes containing 0.25 μl Q5 Hi-Fi DNA polymerase (NEB), 5 μl 10× PCR buffer, 1.25 μl of each primer (10 μM), 0.5 μl dNTP (10 mM) mix, 1 μl genomic DNA and nuclease-free water to volume. Different PCR conditions were carried out for each primer. For ITS and LSU, thermal cycling conditions were as follows: initial denaturation at 98 °C for 30 s; 30 cycles of 98 °C for 10 s, 58 °C for 10 s and 72 °C for 30 s; and a final extension at 72 °C for 2 min. PCR products were checked on 1.2% agarose gels stained with ethidium bromide. For RPB2 (*D. septata*; *D. iloiloensis*), thermal cycling conditions were as follows: initial denaturation at 98 °C for 30 s; 30 cycles of 98 °C for 10 s, 53 °C for 10 s and 72 °C for 30 s; and a final extension at 72 °C for 2 min. For RPB2 (*D. panayensis*; *D. antiqueana*), thermal cycling conditions were as follows: initial denaturation at 98 °C for 30 s; 30 cycles of 98 °C for 10 s, 52.3 °C for 10 s and 72 °C for 30 s; and a final extension at 72 °C for 2 min. For TEF1-α, thermal cycling conditions were as follows: initial denaturation at 98 °C for 30 s; 30 cycles of 98 °C for 10 s, 59 °C for 30 s and 72 °C for 20 s; and a final extension at 72 °C for 2 min. Successful amplicons were purified and sequenced at the Philippine Genome Center – Visayas (University of the Philippines Visayas, Miagao, Iloilo). Newly-generated sequences were assembled and edited in BioEdit v.7.7.1 (Hall 1999) and subjected to BLASTn searches to confirm identity. All sequences were deposited in GenBank (Table 1).

**Table 1.** Taxa used in this study for the analysis of combined ITS, LSU, RPB2 and TEF1-α sequence data and their GenBank accession numbers. The newly-generated sequences are indicated in bold and the ex-type strains are indicated with asterisk (*).

**Taxon**	**Strain Number**	**GenBank Accession Number**			
		**ITS**	**LSU**	**RPB2**	**TEF1-α**
*Aquapteridospora fusiformis*	MFLUCC 18–1606*	MK828652	MK849798	–	MN194056
*A. lignicola*	MFLUCC15–0377*	MZ868774	KU221018	MZ892986	MZ892980
*Distoseptispora adscendens*	HKUCC10820	–	DQ408561	DQ435092	–
*D. amniculi*	MFLUCC17–2129*	MZ868770	MZ868761	MZ892982	–
***D. antiqueana***	UPVMICC 25–0078*	**PZ203859**	**–**	**PZ221688**	**PZ221699**
*D. appendiculata*	MFLUCC18–0259*	MN163009	MN163023	–	MN174866
*D. aqualignicola*	KUNCC 21–10729*	OK341186	ON400845	OP413474	OP413480
*D. aquamyces*	KUNCC 21–10732*	OK341187	OK341199	OP413476	OP413482
*D. aquatica*	MFLUCC18–0646*	MK828648	MK849793	–	MN194052
*D. aquisubtropica*	GZCC 22–0075*	ON527933	ON527941	ON533685	ON533677
*D. arecacearum*	MFLUCC23–0212	OR354399	OR510860	OR481048	OR481045
*D. atroviridis*	GZCC 19–0531	MW133915	MZ227223	–	MZ206155
*D. atroviridis*	GZCC 20–0511*	MZ868772	MZ868763	MZ892984	MZ892978
*D. bambusicola*	GZCC 21–0667*	MZ474873	MZ474872	–	OM272845
*D. bangkokensis*	MFLUCC18–0262*	MZ518205	MZ518206	–	OK067246
*D. bawanglingensis*	SAUCCWZS13‐1*	PQ799295	PQ804721	PQ849357	PQ849363
*D. bawanglingensis*	SAUCCWZS13‐2	PQ799296	PQ804722	PQ849358	PQ849364
*D. cangshanensis*	MFLUCC16–0970*	MG979754	MG979761	–	MG988419
*D. caricis*	CPC 36498*	MN562124	MN567632	MN556805	–
*D. changjiangensis*	SAUCCWZS14‐2	PQ799298	PQ804724	PQ849360	PQ849365
*D. changjiangensis*	SAUCCWZS14‐1*	PQ799297	PQ804723	PQ849359	PQ849366
*D. chengduensis*	CGMCC 3.27439*	PQ067913	PQ067744	–	PQ278565
*D. chiangraiensis*	MFLU 21-0105*	MZ890145	MZ890139	–	MZ892970
*D. chinensis*	GZCC 21–0665*	MZ474871	MZ474867	–	MZ501609
*D. clematidis*	HJAUPC1319	PQ211102	PQ211110	PQ303676	PQ303681
*D. clematidis*	MFLUCC17–2145*	MT310661	MT214617	MT394721	–
*D. combreticola*	GZCC 23‐0729*	PP584670	PP584767	–	PP663310
*D. crassispora*	KUMCC 21–10726*	OK310698	OK341196	OP413473	OP413479
*D. curvularia*	KUMCC 21–10725*	OK310697	OK341195	OP413472	OP413478
*D. cylindricospora*	DLUCC1906*	OK491122	OK513523	–	OK524220
*D. daanyuanensis*	SAUCC12326‐1*	PV670056	PV670405	–	PV708057
*D. daanyuanensis*	SAUCC12326‐2	PV670057	PV670406	–	PV708058
*D. davidalangii*	UESTCC 23.0473	PQ191048	PQ184723	PQ379953	PQ346499
*D. davidalangii*	UESTCC 24.0236*	PQ189781	PQ184736	PQ380000	PQ346519
*D. davidii*	GZCC 24-0091*	PV820360	PV856177	–	–
*D. dehongensis*	KUMCC 18–0090*	MK085061	MK079662	–	MK087659
*D. dinghuensis*	ZHKUCC 23-0958	PQ037957	PQ037956	PQ035180	PQ035181
*D. dipterocarpi*	MFLUCC22–0104*	OP600053	OP600052	OP595140	–
*D. dujuanhuensis*	KUNCC:23-13772	PQ845849	PV536297	PX233767	PX238245
*D. effusa*	GZCC 19–0532*	MW133916	MZ227224	–	MZ206156
*D. eleiodoxae*	MFLUCC23–0214*	OR354398	OR510859	OR481047	OR481044
*D. euseptata*	DLUCCS2024	MW081540	MW081545	MW084996	MW084994
*D. euseptata*	MFLUCC20–0154*	MW081539	MW081544	MW151860	–
*D. foveolata*	MFLUCC20–0091*	MT232713	MT232718	MT232881	MT232880
*D. fasciculata*	KUMCC 19–0081*	MW286501	MW287775	–	MW396656
*D. fasciculata (=D. mengsongensis)*	HJAUP C2126*	OP787876	OP787874	–	OP961937
*D. fluminicola*	DLUCC 0391	MG979755	MG979762	–	MG988420
*D. fluminicola*	DLUCC 0999	MG979756	MG979763	–	MG988421
*D. fujianensis*	HJAUPC2509*	PQ211095	PQ211103	PQ303679	PQ303682
*D. fujianensis*	HJAUPC2513	PQ211098	PQ211106	PQ303680	PQ303683
*D. fusiformis*	GZCC 20–0512*	MZ868773	MZ868764	MZ892985	MZ892979
*D. ganzhouensis*	HJAUPC1090*	PQ211100	PQ211108	–	PQ303687
*D. gasaensis*	HJAUPC2034*	OQ942896	OQ942891	–	OQ944455
*D. gelatinosa*	MFLU 24-0292*	PQ570855	PQ570872	–	–
*D. guanshanensis*	HJAUPC1063*	OQ942894	OQ942898	OQ944458	OQ944452
*D. guanxiensis*	GZCC 26-0131	PX984883	PX984885	PZ013919	PZ013918
*D. guanxiensis*	GZCC 26-0130 T	PX889843	PX984884	–	–
*D. guizhouensis*	GZCC 21–0666*	MZ474868	MZ474869	MZ501611	MZ501610
*D. guttulata*	MFLU 17–0852*	MF077543	MF077554	–	MF135651
*D. hainanensis*	GZCC 22-2047*	OR427328	OR438894	OR449119	OR449122
*D. heptapleuricola*	CGMCC 3.27740*	PQ189783	PQ184738	PQ380001	PQ346520
*D. hongheensis*	KUNCC:23-14299*	PQ845851	PV536299	PX233769	PX238247
*D. hyalina*	MFLUCC 17-2128*	MZ868769	MZ868760	MZ892981	MZ892976
*D. hydei*	MFLUCC 20-0481*	MT734661	MT742830	–	–
***D. iloiloensis***	UPVMICC 25–0024*	**PZ203857**	**PZ208527**	**–**	**PZ221697**
*D. jianfenglingensis*	SAUCC WZS65-3*	PQ799299	PQ804725	PQ849361	PQ849367
*D. jianfenglingensis*	SAUCC WZS65-4	PQ799300	PQ804726	PQ849362	PQ849368
*D. jingdongensis*	KUNCC:23-14297*	PQ845853	PV536301	PX233771	PX238249
*D. jinghongensis*	HJAUP C2120*	OQ942897	OQ942893	–	OQ944456
*D. keviniana*	GZCC 24-0289*	PV820358	–	–	–
*D. keviniligustrina*	UESTCC 23.0471	PQ191052	PQ184727	PQ379955	PQ346501
*D. lancangjiangensis*	DLUCC 1864*	MW723055	MW879522	MW882260	–
*D. lanceolatispora*	GZCC 22-2045*	OR427329	OR438895	OR449120	OR449123
*D. leonensis*	HKUCC 10822	–	DQ408566	DQ435089	–
*D. licualae*	MFLUCC 14-1163A*	ON650686	ON650675	–	ON734007
*D. licualae*	MFLUCC 14-1163B*	ON650687	ON650676	–	ON734008
*D. lignicola*	GZCC 19-0529	MW133911	MZ227219	–	MZ206152
*D. lignicola*	HFJAU 0705*	MK828651	MK849797	–	–
*D. linchunlingensis*	SAUCC1738201*	PZ100104	PZ112124	PZ121388	PZ121394
*D. linchunlingensis*	SAUCC1738202	PZ100105	PZ112125	PZ121389	PZ121395
*D. liupanshuiensis*	GZCC 23-0730*	PP584669	PP584766	–	PP663309
*D. longiconidiophora*	GZCC 25-0679*	PX860093	PX889838	PX890981	PX890982
*D. longiconidiophora*	GZCC 25-0678	PX860094	PX889839	–	–
*D. longispora*	HFJAU 0705*	MH555359	MH555357	–	–
*D. longissima*	GMBC5339*	PV932968	PV932987	PX373356	PX392330
*D. longissima*	GMBC5340	PV932969	PV932988	PX373357	PX392331
*D. longnanensis*	HJAUP C1040*	OQ942887	OQ942886	–	OQ944451
*D. mangrovei*	ZHKUCC 25-0793	PX806213	PX811045	PX965873	PX965874
*D. martinii*	CGMCC 3.18651*	KU999975	KX033566	–	–
*D. meilingensis*	JAUCC 4727*	OK562390	OK562396	–	OK562408
*D. meilingensis*	JAUCC 4728*	OK562391	OK562397	–	OK562409
*D. menghaiensis*	HJAUP C2045*	OQ942890	OQ942900	–	–
*D. menglunensis*	HJAUP C2170	OQ942899	OQ942888	OQ944461	OQ944457
*D. monospora*	HKAS:145690*	PQ898696	PQ898699	PV001672	PV001669
*D. motuoensis*	KUNCC24-17628*	PP600327	PP621731	–	PP639546
*D. muchuanensis*	CGMCC 3.27444*	PQ067919	PQ067750	–	PQ278571
*D. multiseptata*	MFLU 17-0856	MF077544	MF077555	MF135644	MF135652
*D. multiseptata*	MFLUCC 15-0609*	KX710145	KX710140	–	MF135659
*D. nabanheensis*	HJAUP C2003*	OP787873	OP787877	–	OP961935
*D. nanchangensis*	HJAUP C1074*	OQ942889	OQ942895	OQ944460	OQ944454
*D. nanpingensis*	HJAUP C2517*	PQ211096	PQ211104	PQ303678	–
*D. narathiwatensis*	MFLUCC 23-0216	OR354400	OR510861	OR481049	OR481046
*D. neorostrata*	MFLUCC 18-0376*	MN163008	MN163017	–	–
*D. nonrostrata*	KUNCC 21-10730*	OK310699	OK341198	OP413475	OP413481
*D. obclavata*	MFLUCC 18-0329*	MN163012	MN163010	–	–
*D. obpyriformis*	MFLUCC 17-1694*	–	MG979764	MG988415	MG988422
*D. obpyriformis*	DLUCC 0867	MG979757	MG979765	MG988416	MG988423
*D. olivaceoviridis*	MFLU 24-0290	PQ568144	PQ569325	–	–
*D. pachyconidia*	KUMCC 21-10724*	OK310696	OK341194	OP413471	OP413477
*D. palmarum*	MFLUCC 18-1446*	MK085062	MK079663	MK087670	MK087660
***D. panayensis***	UPVMICC 25–0088*	**PZ203858**	**PZ208528**	**PZ221687**	**PZ221698**
*D. phangngaensis*	MFLUCC 16-0857*	MF077545	MF077556	–	MF135653
*D. phangngaensis (=D. thailandica)*	MFLUCC 16–0270*	MH275060	MH260292	–	MH412767
*D. phragmiticola*	GUCC 220201*	OP749887	OP749880	OP752699	OP749891
*D. phragmiticola*	GUCC 220202*	OP749888	OP749881	OP752700	OP749892
*D. polyblasta*	GZCC 25-0526*	PV820359	PV856176	–	–
*D. pseudoaquisubtropica*	GZCC 24-0061*	PV820361	PV856178	–	–
*D. pulchra*	HKAS:132106*	PQ427204	PQ576433	PX233824	PX238335
*D. quinqueseptata*	GMBC5341*	PV932966	PV932985	PX373358	PX392328
*D. quinqueseptata*	GMBC5342	PV932967	PV932986	PX373359	PX392329
*D. rayongensis*	MFLUCC 18-0415*	MH457172	MH457137	MH463255	MH463253
*D. rayongensis*	MFLUCC 18-0417	MH457173	MH457138	MH463256	MH463254
*D. rostrata*	MFLUCC 16-0969*	MG979758	MG979766	MG988417	MG988424
*D. rostrata*	DLUCC 0885	MG979759	MG979767	–	MG988425
*D. saprophytica*	MFLUCC 18-1238*	MW286506	MW287780	MW504069	MW396651
*D. septata*	GZCC 22-0078*	ON527939	ON527947	ON533690	ON533683
*D. septata*	UPVMICC 25–0100	PZ203856	PZ208526	PZ221686	PZ221696
*D. sichuanensis*	KUNCC 23-15519*	PP584672	PP584769	–	PP663312
*D. sinensis*	HJAUP C2044*	OP787878	OP787875	–	OP961936
*D. solitaria*	GZCC 24-0038*	PV820362	PV856179	–	–
*D. songkhlaensis*	MFLUCC 18-1234*	MW286482	MW287755	–	MW396642
*D. suae*	CGMCC 3.24262*	OQ874968	OQ732679	OQ870341	OR367670
*D. submersa*	MFLUCC 16-0946*	MG979760	MG979768	MG988418	MG988426
*D. subtropica*	HJAUP C2528*	PQ211099	PQ211107	PQ303677	PQ303684
*D. subtropica*	HJAUP C2535	PQ211097	PQ211105	–	PQ303685
*D. suoluoensis*	MFLUCC 17–0224*	MF077546	MF077557	–	MF135654
*D. suoluoensis*	MFLUCC 17–0854	MF077547	MF077558	MZ945510	–
*D. tectonae*	MFLUCC 12–0291*	KX751711	KX751713	KX751708	KX751710
*D. tectonae*	MFLU 20–0262	MT232714	MT232719	–	–
*D. tectonigena*	MFLUCC 12–0292*	KX751712	KX751714	KX751709	–
*D. terrestris*	HJAUP C2539*	PV448667	PV450538	–	PV469764
*D. thysanolaenae*	KUN–HKAS 102247*	MK045851	MK064091	–	MK086031
*D. thysanolaenae*	KUN–HKAS 112710	MW723057	MW879524	–	MW729783
*D. tongrensis*	GMBC5343*	PV932970	PV932989	PX373360	–
*D. tongrensis*	GMBC5344	PV932971	PV932990	PX373361	–
*D. trichospora*	ZHKUCC 24-1259	PX369568	PX693004	–	–
*D. tropica*	GZCC 22–0076*	ON527935	ON527943	ON533687	ON533679
*D. uncariicola*	UESTCC 24.0229*	PQ189784	PQ184739	PQ380002	PQ346521
*D. vaginae*	GZCC 25-0527*	PV820363	PV856180	–	–
*D. velvetica*	GZCC 24-0286*	PV820364	PV856181	–	–
*D. verrucosa*	GZCC 20–0434*	MZ868771	MZ868762	MZ892983	MZ892977
*D. wuyishanensis*	HJAUP C2515 T	PV448666	PV450537	PV469759	PV469763
*D. wuzhishanensis*	GZCC 22–0077*	ON527938	ON527946	–	ON533682
*D. xinganensis*	SAUCC1835501*	PZ100106	PZ112126	PZ121390	PZ121396
*D. xinganensis*	SAUCC1835502	PZ100107	PZ112127	PZ121391	PZ121397
*D. xinpingensis*	KUNCC 22–12667*	OQ874970	OQ732681	OQ870340	OR367671
*D. xishuangbannaensis*	KUMCC 17–0290*	MH275061	MH260293	MH412754	MH412768
*D. yichunensis*	HJAUP C1065*	OQ942885	OQ942892	OQ944459	OQ944453
*D. yongxiuensis*	JAUCC 4725*	OK562388	OK562394	–	OK562406
*D. yunjushanensis*	JAUCC 4724*	OK562392	OK562398	–	OK562410
*D. yunjushanensis*	HJAUPC1307	PQ211101	PQ211109	PQ303675	PQ303686
*D. yunnanensis*	MFLUCC20–0153*	MW081541	MW081546	MW151861	MW084995
*D. zhejiangensis*	HJAUP C2588*	PV448668	PV450539	–	PV469765
*D. zunyiensis*	KUNCC:24-18628*	PV918496	PV918482	–	PX048436

### Phylogenetic analyses

Sequences generated in this study were queried in the NCBI GenBank database using BLASTn. Closely-related taxa were selected, based on the sequence similarity, query coverage and recent phylogenetic studies of He et al. (2026) and Tian et al. (2026). Corresponding reference sequences were retrieved from GenBank for inclusion in the phylogenetic dataset. The aligned datasets (ITS, LSU, RPB2, TEF1-α) were analysed separately and as a concatenated matrix. Sequence alignments were performed using MAFFT v.7 (http://mafft.cbrc.jp/alignment/server/; Katoh et al. (2019)) and manually adjusted in BioEdit where necessary. Phylogenetic analyses were conducted through the CIPRES Science Gateway v.3.3 (https://phylo.org/portal2/; Miller et al. (2010)) using Maximum Likelihood (ML) and Bayesian Inference (BI). ML analyses were performed with RAxML v.8.2.8 (Stamatakis 2006; Stamatakis et al. 2008) implemented in the RAxML-HPC2 on XSEDE tool. All model parameters were estimated by RAxML, with site-rate heterogeneity approximated using 25 discrete rate categories and the final tree was inferred under the GTRGAMMA+I substitution model. BI analyses were performed using MrBayes v.3.2.7a (Ronquist and Huelsenbeck 2003). The best-fit nucleotide substitution model for each dataset was selected using MrModelTest v.2.2 (Nylander 2004) and the GTR+I+G model was selected for all individual loci and the concatenated dataset. Two independent runs, each consisting of four Markov chains, were conducted for 10 million generations, with trees sampled every 100 generations, resulting in 100,000 sampled trees. The first 25% of sampled trees were discarded as burn-in and the remaining 75,000 trees were used to construct a 50% majority-rule consensus tree. Bayesian posterior probabilities (PP) were calculated from the post-burn-in trees to assess branch support. Phylogenetic trees were visualised in FigTree v.1.4.4 (https://tree.bio.ed.ac.uk/software/figtree/) and finalised in Adobe Illustrator 2026 (Adobe 2026). The concatenated alignment is available in Figshare ( https://doi.org/10.6084/m9.figshare.32747727).

## Results

### Phylogenetic analyses

**Figure 1.**
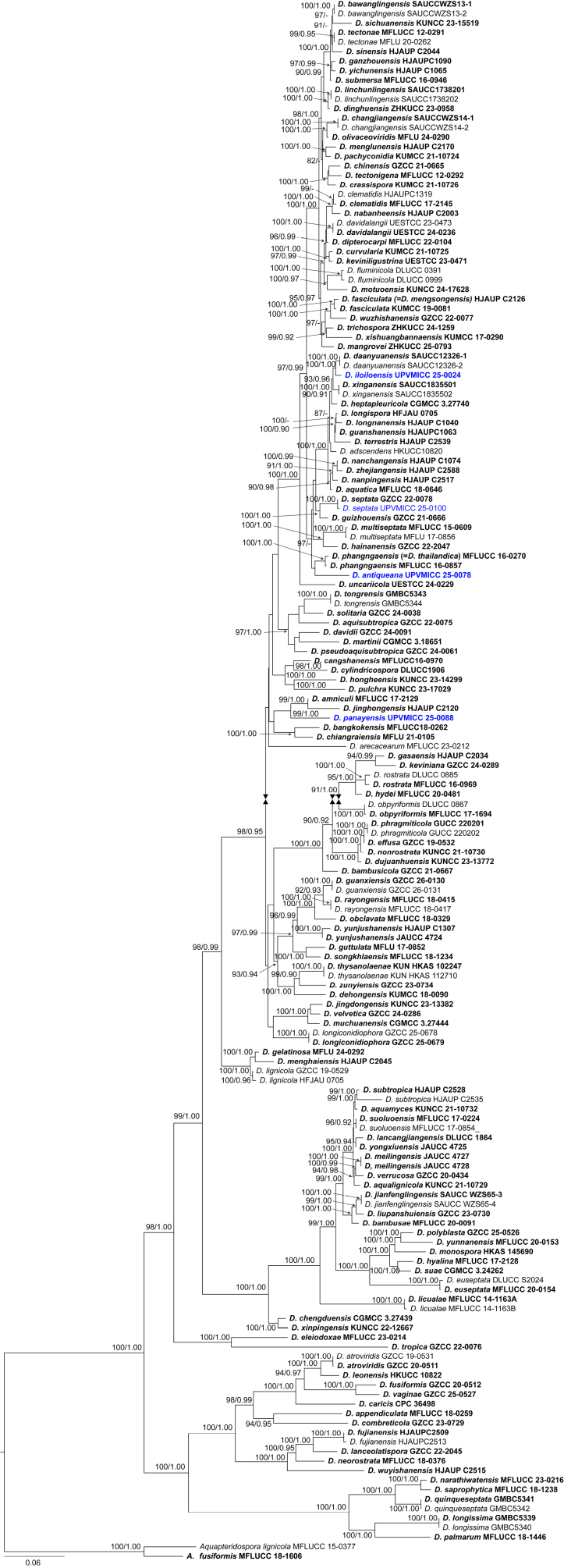
Phylogenetic tree generated from Maximum Likelihood analysis, based on ITS, LSU, RPB2 and TEF1-α sequence data for the species from *Distoseptispora*. Bootstrap support values for ML higher than 75% and Bayesian posterior probabilities (BYPP) greater than 0.95 are indicated above the nodes in this order. The new isolates are represented in blue. The ex-type strains are indicated in bold. The tree is rooted to *Aquapteridospora fusiformis* (MFLUCC 18–1606) and *A. lignicola* (MFLUCC15–0377). Bar = 0.07 estimated number of nucleotide substitutions per site per branch.

The combined multigene analyses of *Distoseptispora* included one hundred and sixty-four taxa, with *Aquapteridospora fusiformis* (MFLUCC 18–1606) and *A. lignicola* (MFLUCC15–0377) serving as outgroup taxa (Table 1, Fig. 1). The analysed dataset, after trimming, comprised a total of 3861 characters including gaps (ITS = 417 bp; LSU = 1392 bp; RPB2 = 1029 bp; TEF1-α = 1023 bp). The ML analysis for the combined dataset provided the best scoring tree (Fig. 1) with a final ML optimisation likelihood value of -45703.083 (ln). The matrix had 3378 columns, 1804 distinct patterns, 1227 parsimony-informative, 265 singleton sites and 1886 constant sites. Parameters for the GTRGAMMA+I of the combined ITS, LSU, RPB2 and TEF1-α dataset are as follows: estimated base frequencies; A = 0.238, C = 0.269, G = 0.280, T = 0.214; substitution rates AC = 1.44573, AG = 4.06967, AT = 1.44573, CG = 1.0, CT = 8.18233, GT = 1.0; gamma distribution shape parameter α = 0.741.

In the multi-locus phylogenetic analysis, three novel *Distoseptispora* species, *D. antiqueana*, *D. iloiloensis* and *D. panayensis* grouped with the other *Distoseptispora* species (Fig. 1). In addition, the strain UPVMICC 25–0100 clustered with the holotype of *D. septata* GZCC 22-0078 with strong bootstrap support (100% MLBS, 1.00 BYPP).

### Taxonomy

#### Distoseptispora antiqueana

Taxon classificationFungiDistoseptisporalesDistoseptisporaceae

Bagacay, E.B.G. Jones & M.S. Calabon sp. nov.

173CA2E3-D5CA-5BDB-9461-99AF85DEAF6F

Fig. 2

##### Etymology.

Named after Antique Province, Philippines, where the fungus was first observed.

**Figure 2.**
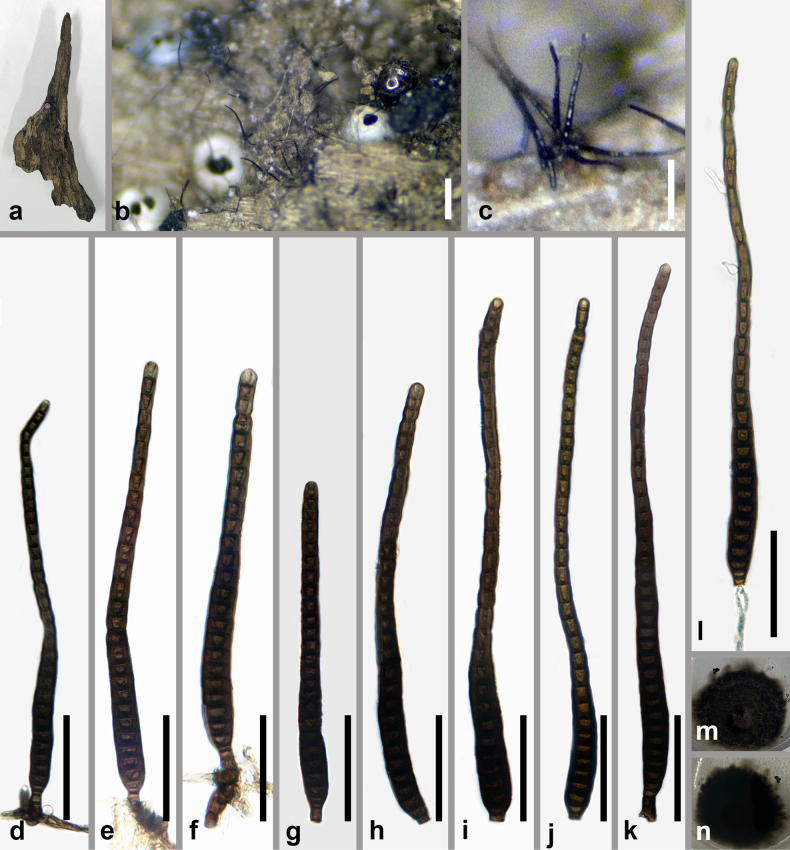
*Distoseptispora antiqueana* (UPVMICC 25–0078, holotype). **a**. Host; **b, c**. Colonies on the host surface; **d–f**. Conidiophores and conidiogenous cells bearing conidia; **g–k**. Conidia; **l**. Germinating conidium; **m, n**. Colonies on PDA, **m**. From above, **n**. From reverse. Scale bars: 500 μm (**b**); 200 μm (**c**); 50 μm (**d–l**).

##### Holotype.

UPVMI 25–0078.

##### Description.

***Saprobic*** on submerged decaying wood in intertidal habitat. **Sexual morph**: Undetermined. **Asexual morph**: ***Colonies*** superficial, effuse, gregarious, brown or dark brown, hairy. ***Mycelium*** mostly immersed, composed of branched, septate, smooth, pale brown to brown hyphae. ***Conidiophores*** 12–35 × 7–11 μm (x¯ = 21.5 × 8.9 μm, n = 15), macronematous, mononematous, brown to dark brown, solitary, 2–4-septate, erect, straight, or flexuous, unbranched, single or in groups of 2 or 3, smooth, cylindrical, truncate at the apex, slightly constricted at septa. ***Conidiogenous cells*** holoblastic, monoblastic, integrated, terminal, determinate, smooth, cylindrical. ***Conidia*** 139–268 × 9–16 (x¯ = 201.6 × 12.9 μm, n = 30) acrogenous, solitary, dry, obclavate, cylindrical or rostrate, elongated, smooth-walled, straight or slightly curved, up to 39-distosepta, guttulate, olivaceous to dark brown, mostly slightly constricted at septa, tapering towards the rounded apex, truncate at the base. Conidial secession schizolytic.

##### Culture characteristics.

Conidia germinated on potato dextrose agar supplemented with sea water and produced germ tubes within 24 h. Colonies grown on PDA, circular, mycelium flat, dense, reaching 30–35 mm diam. after 30 days of incubation at 25 °C, grey or brown, reverse-side dark brown.

##### Material examined.

Philippines, Antique, Anini-y, Nogas Island, on submerged wood from the intertidal zone, 30 September 2023, Jan Felnesh Exe Bagacay, NOG-005 (UPVMI 25–0078), living culture UPVMICC 25–0078.

##### GenBank accession numbers.

ITS = PZ203859, RPB2 = PZ221688, TEF1-α = PZ221699.

##### Notes.

In the multi-locus phylogenetic analyses, *Distoseptispora antiqueana*UPVMICC 25–0078 formed a well-supported, distinct lineage basal to a strongly-supported clade comprising *D. thailandica* (MFLUCC 16-0270), *D. phangngaensis* (MFLUCC 16-0857), *D. hainanensis* (GZCC 22-2047), *D. multiseptata* (MFLUCC 15-0609),and other closely-related taxa (100 MLBS, 1.00 BYPP; Fig. 1). BLASTn comparisons further support its phylogenetic distinction. The ITS sequence shows highest similarity to *D. phangngaensis*MFLUCC 16-0857 (99.15%). However, protein-coding loci reveal lower similarity values: RPB2 shows 96.88% similarity to *D. nanpingensis* (HJAUP C2517) and TEF1-α shows 94.69% similarity to *D. jinghongensis* (HJAUP C2120). Pairwise nucleotide comparisons further support the recognition of *Distoseptispora antiqueana* as a distinct species, differing from *D. phangngaensis* by 0.34% (2/588 bp, ITS) and 7.56% (73/965 bp, TEF1-α) sequence divergence. The introduction of *Distoseptispora antiqueana*, as with all novel taxa described in this study, follows the integrative species delimitation approach of Jeewon and Hyde (2016), whereby species are recognised, based on concordant phylogenetic evidence, nucleotide differences across informative loci and diagnostic morphological characters. *Distoseptispora antiqueana* differs from *D. phangngaensis* by having shorter, but wider conidiophores (12–35 × 7–11 μm vs. 18–30(–40) × 4.3–6.5 μm), shorter and narrower conidia (139–268 × 9–16 μm vs. 165–350 × 14–19 μm) (Yang et al. 2018). The observed interspecific divergence in the protein-coding genes, together with its independent phylogenetic position and morphological differences support its designation as a distinct species.

This represents the second confirmed record of *Distoseptispora* from a marine habitat, isolated from submerged decaying wood in a mangrove ecosystem; the first such record is *D. mangrovei* reported from China (Li et al. 2026). Members of the genus are predominantly reported from freshwater and terrestrial environments; thus, this finding expands the known ecological range of the genus into marine-associated substrates (Calabon et al. 2022; Ma et al. 2022; Hu et al. 2023; Shen et al. 2024; Liao et al. 2025).

#### Distoseptispora iloiloensis

Taxon classificationFungiDistoseptisporalesDistoseptisporaceae

Bagacay, Candole, E.B.G. Jones & M.S. Calabon sp. nov.

ABDCD0B9-A0DA-5E67-B3E1-66F816F81099

Fig. 3

##### Etymology.

Named after Iloilo Province, Philippines, where the fungus was first observed.

**Figure 3.**
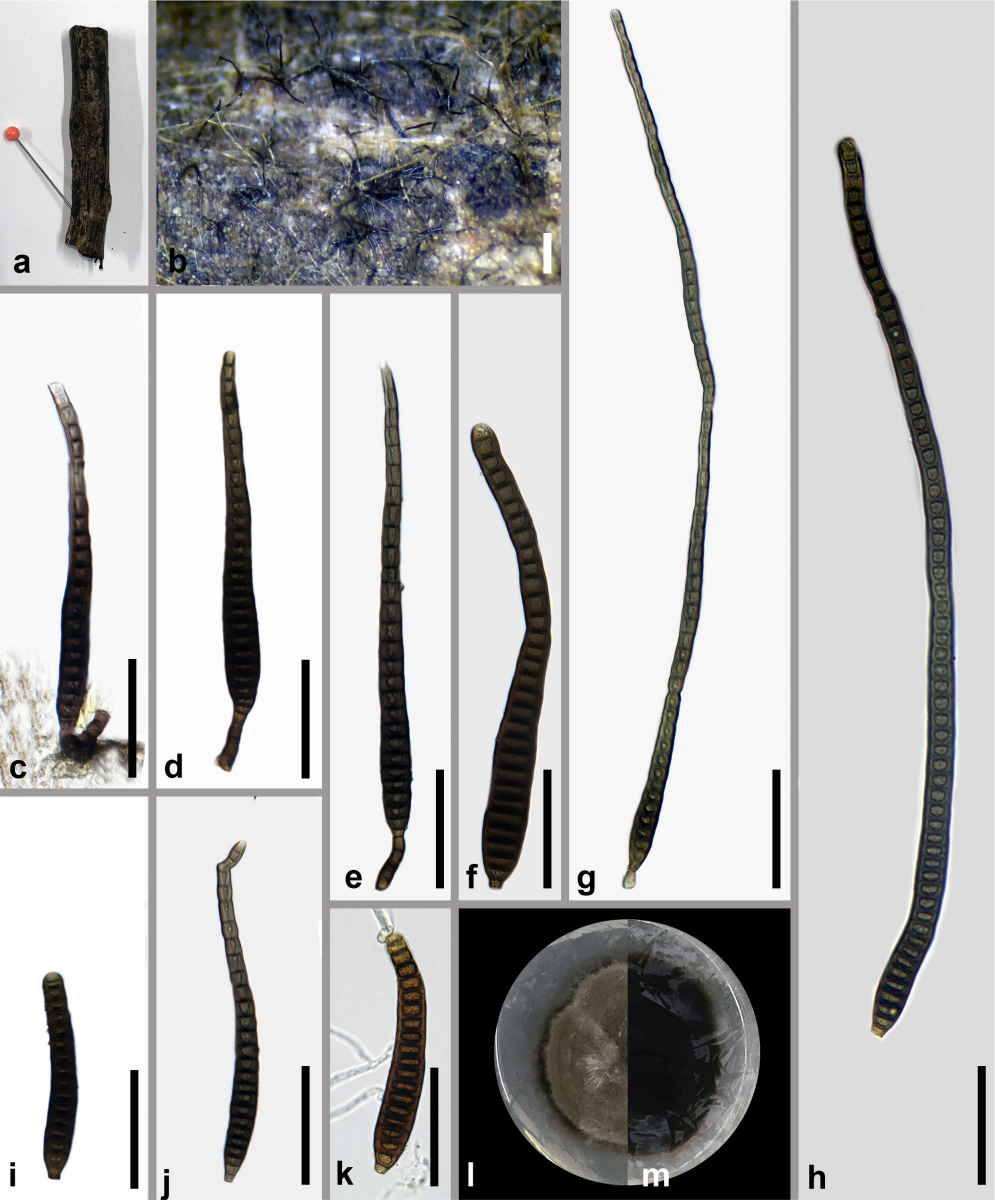
*Distoseptispora iloiloensis* (UPVMI 25–0024, holotype). **a**. Host; **b**. Colonies on the host surface; **c–e**. Conidiophores and conidiogenous cells bearing conidia; **f–j**. Conidia; **k**. Germinating conidium; **l, m**. Colonies on PDA, **l**. From above, **m**. From reverse. Scale bars: 200 μm (**b**); 50 μm (**d–k**).

##### Holotype.

UPVMI 25–0024.

##### Description.

***Saprobic*** on submerged decaying wood in freshwater habitat. **Sexual morph**: Undetermined. **Asexual morph**: ***Colonies*** superficial, effuse, gregarious, brown or dark brown, hairy. ***Mycelium*** mostly immersed, composed of branched, septate, smooth, pale brown to brown hyphae. ***Conidiophores*** 14–34 × 4–8 μm (x¯ = 25.1 × 6.3 μm, n = 10), macronematous, mononematous, brown to dark brown, solitary, 2–4-septate, erect, straight or flexuous, unbranched, single or in groups of 2 or 3, smooth, cylindrical, truncate at the apex, slightly constricted at septa. ***Conidiogenous cells*** holoblastic, monoblastic, integrated, terminal, determinate, smooth, cylindrical. ***Conidia*** (82–)102–200(–385) × 6–19 µm (x¯ = 158.4 × 13.7 µm, n = 25) acrogenous, solitary, dry, obclavate, cylindrical or rostrate, elongated, smooth-walled, straight or slightly curved, up to 39-distoseptate, guttulate, olivaceous to dark brown, mostly slightly constricted at septa, tapering towards the rounded apex, truncate at the base. Conidial secession schizolytic.

##### Culture characteristics.

Conidia germinated on potato dextrose agar and produced germ tubes within 24 h. Colonies grown on PDA, circular, mycelium flat, dense, reaching 25–30 mm diam. after 30 days of incubation at 25 °C, grey or brown, reverse-side dark brown.

##### Material examined.

Philippines, Iloilo, Miagao, Tinagong Dagat Lake, on submerged wood from a stream, 23 September 2023, Gerlie Mae Candole, TGFW12 (UPVMI 25–0024), living culture UPVMICC 25–0024.

##### GenBank accession numbers.

ITS = PZ203857, LSU = PZ208527, TEF1-α = PZ221697

##### Notes.

*Distoseptispora iloiloensis* forms a well-supported clade with *D. daanyuanensis* (SAUCC12326-1, SAUCC12326-2), based on combined ITS–LSU–RPB2-TEF1-α sequence data (100% MLBS, 1.00 BYPP; Fig. 1). Both taxa conform to the generic circumscription of *Distoseptispora* in producing holoblastic, monoblastic, acrogenous conidiogenous cells and distoseptate, obclavate to rostrate conidia. Morphologically, the two species are readily separable by conidial dimensions and septation patterns. *Distoseptispora daanyuanensis* is characterised by exceptionally long and highly septate conidia (23.1–696.2 × 9.3–17.9 μm; 4–92 distosepta), representing the broadest range in both size and septation (Liu et al. 2025). In contrast, *D. iloiloensis* is distinguished by comparatively smaller conidia ((82–)102–200(–385) × 6–19 μm) with up to 39 distosepta, generally lacking apical percurrent regeneration. Pairwise nucleotide comparisons further support their distinction wherein *D. iloiloensis* differs from *D. daanyuanensis* by 1.40% (8/573 bp, ITS), 1.78% (15/843 bp, LSU) and 0.78% (7/892 bp, TEF1-α) sequence divergence. Ecologically, the taxa occupy discrete substrates: *D. daanyuanensis* is reported from decaying wood in peat swamp forest and *D. iloiloensis* from submerged wood in freshwater habitat (Liu et al. 2025). This ecological segregation, coupled with subtle, yet stable morphological differences, suggests niche specialisation within this phylogenetically cohesive lineage.

#### Distoseptispora panayensis

Taxon classificationFungiDistoseptisporalesDistoseptisporaceae

Bagacay, E.B.G. Jones & M.S. Calabon sp. nov.

CCB84D1C-4774-5371-99F2-929C30BFA1B2

Fig. 4

##### Etymology.

Named after Panay Island, Philippines, where the fungus was first observed.

**Figure 4.**
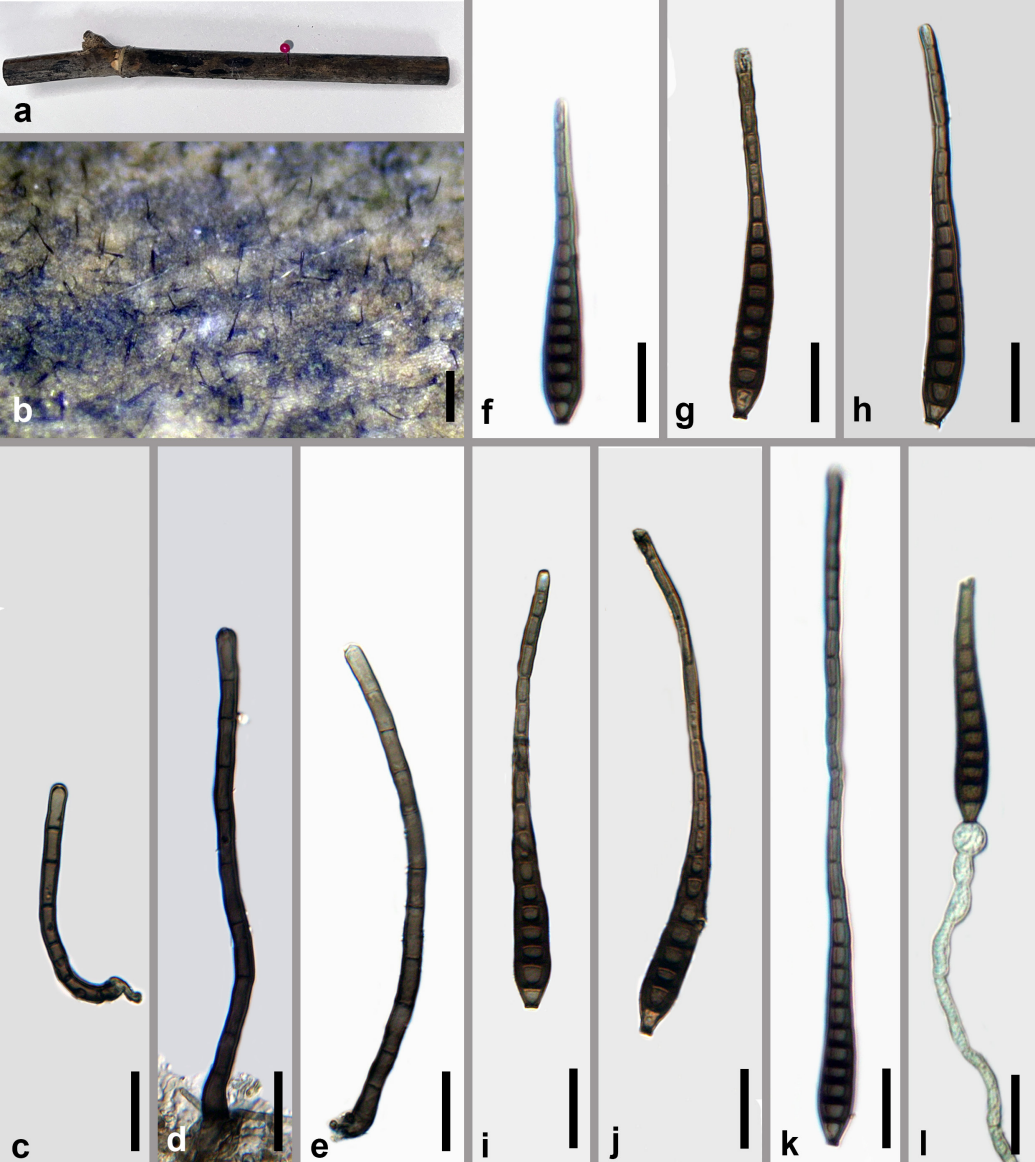
*Distoseptispora panayensis* (UPVMI 25–0100, holotype). **a**. Host; **b**. Colonies on the host surface; **c–e**. Conidiophores; **f–k**. Conidia; **l**. Germinating conidium. Scale bars: 100 μm (**b**); 20 μm (**c–l**).

##### Holotype.

UPVMI 25–0100.

##### Description.

***Saprobic*** on submerged decaying wood in freshwater habitat. **Sexual morph**: Undetermined. **Asexual morph**: Hyphomycetous. ***Colonies*** superficial, effuse, scattered or in small groups, hairy, dark brown. ***Mycelium*** partly immersed, comprised of branched, septate, smooth, pale brown to brown hyphae. ***Conidiophores*** 66–134 × 4–8 μm (x¯ = 111.7 × 5.2 μm, n = 15), macronematous, mononematous, solitary, brown, 6–10-septate, erect, straight or slightly flexuous, unbranched, smooth, cylindrical, rounded and darkened at the apex, sometimes elongating percurrently. ***Conidiogenous cells*** holoblastic, monoblastic, integrated, terminal, brown, determinate, cylindrical. ***Conidia*** 82–174 × 7–12 μm (x¯ = 117.8× 9.4 μm, n = 30) acrogenous, solitary, dry, obclavate, rostrate, elongated, smooth-walled, straight or slightly curved, 13–23-distoseptate, olivaceous brown to greyish-brown, tapering towards the apex, truncate and darkened at the base. Conidial secession schizolytic.

##### Culture characteristics.

Conidia germinated on potato dextrose agar and produced germ tubes within 10 h. Colonies grown on PDA, circular, mycelium flat, dense, reaching 35–40 mm diam. after 30 days of incubation at 25 °C, grey or brown, reverse-side dark brown.

##### Material examined.

Philippines, Iloilo, Miagao, Busay-lapad Falls, on submerged decaying wood from a stream, 26 January 2024, Jan Felnesh Exe Bagacay, BL-F-004 (UPVMI 25–0100), living culture UPVMICC 25–0100.

##### GenBank accession numbers.

ITS = PZ203858, LSU = PZ208528, RPB2 = PZ221687, TEF1-α = PZ221698.

##### Notes.

Multi-locus phylogenetic analyses place *D. panayensis*UPVMICC 25–0100 in a well-supported clade with *D. amniculi*MFLUCC 17-2129 and *D. jinghongensis*HJAUP C2120 (99% MLBS, 1.00 BYPP, Fig. 1). Pairwise nucleotide comparisons further substantiate its distinction: *D. panayensis* differs from *D. amniculi* by 2.9% (16/551 bp, ITS) and 0.8% (7/875 bp, LSU) and 7.53% (80/1063 bp, TEF1- α). Morphologically, *D. panayensis* is characterised by relatively long and moderately broad conidiophores (66–134 × 4–8 μm, x¯ = 111.7 × 5.2 μm) that are 6–10-septate and occasionally show percurrent elongation. It differs from *D. amniculi*, which possesses longer, but distinctly narrower conidiophores (90–180 × 3–4.5 μm) (Yang et al. 2021). *Distoseptispora panayensis* produces conspicuously long, obclavate-rostrate conidia (82–174 × 7–12 μm, x¯ = 117.8 × 9.4 μm) with 13–23 distosepta and overlapping, but generally surpassing that of *D. amniculi* ((7–)12–24-septate; 85–167 × 9–11.8 μm). Ecologically, *D. panayensis* and *D. amniculi* are freshwater taxa occurring on submerged decaying wood (Yang et al. 2021). Pairwise nucleotide comparisons between *D. panayensis* and *D. jinghongensis* revealed sequence differences of 4.84% (29/599 bp) in the ITS region, 3.97% (23/579 bp) in LSU and 7.38% (71/962 bp) in TEF1-α. Morphologically, *D. panayensis* differs by having longer and wider conidiophores (66–134 × 4–8 μm vs. 54.6–94.6 × 3.6–4 μm), more septate conidiophores (6–10 vs. 4–7 septa) and considerably longer conidia (82–174 × 7–12 μm vs. 56.4–127.3 × 7.3–10.9 μm) with a greater number of distosepta (13–23 vs. 7–17) (Hu et al. 2023). Furthermore, *D. jinghongensis* was observed on dead branches of an unidentified broadleaf tree. The consistent morphological and multi-locus phylogenetic distinctions support the establishment of *D. panayensis* as a novel species.

#### Distoseptispora septata

Taxon classificationFungiDistoseptisporalesDistoseptisporaceae

Jian Ma & Y.Z. Lu, J. Fungi 8(no. 1202): 15 (2022)

622AC26C-E53C-5C0C-839C-D1451913B006

Fig. 5

##### Description.

***Saprobic*** on submerged decaying wood in freshwater habitat. **Sexual morph**: Undetermined. **Asexual morph**: ***Colonies*** superficial, effuse, gregarious, brown or dark brown, hairy. ***Mycelium*** mostly immersed, composed of branched, septate, smooth, pale brown to brown hyphae. ***Conidiophores*** up to 20 μm, macronematous, mononematous, brown to dark brown, solitary, 2–3-septate, erect, straight or flexuous, unbranched, single or in groups of 2, smooth, cylindrical, truncate at the apex, slightly constricted at septa. ***Conidiogenous cells*** holoblastic, monoblastic, integrated, terminal, determinate, smooth, cylindrical. ***Conidia*** 129–220 × 9–15 μm (x¯ = 159.5 × 12.4 μm, n = 30) acrogenous, solitary, dry, obclavate, cylindrical or rostrate, elongated, smooth-walled, straight or slightly curved, up to 46-distoseptate, guttulate, olivaceous to dark brown, mostly slightly constricted at septa, tapering towards the rounded apex, truncate at the base. Conidial secession schizolytic.

**Figure 5.**
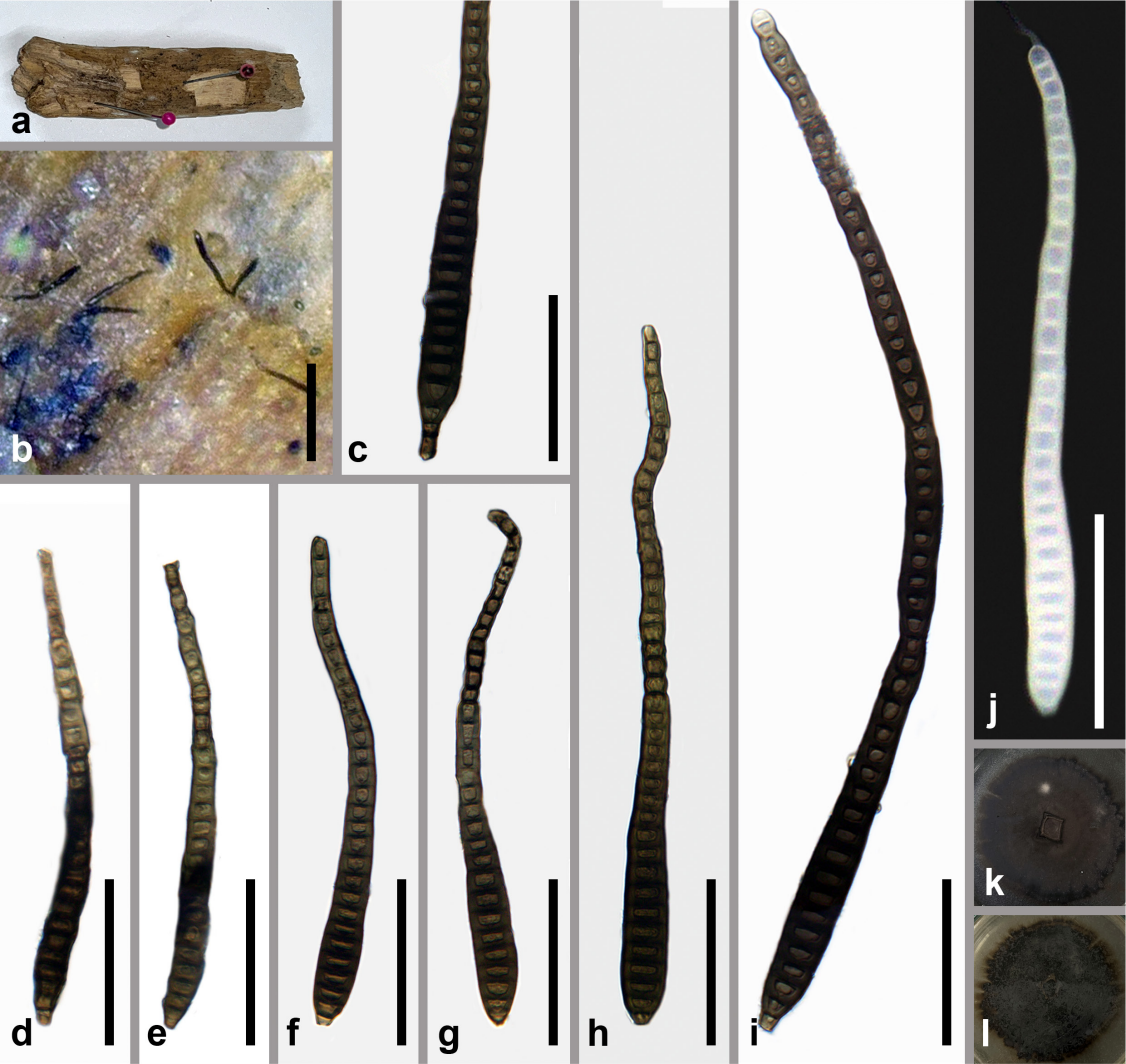
*Distoseptispora septata* (UPVMI 25–0088). **a** Host; **b**. Colonies on the host surface; **c**. Conidiophores and conidiogenous cells bearing conidia; **d–i**. Conidia; **j**. Germinating conidium; **k, l**. Colonies on PDA; **k**. From above; **l**. From reverse. Scale bars: 200 μm (**b**); 50 μm (**c–j**).

##### Culture characteristics.

Conidia germinated on potato dextrose agar and produced germ tubes within 24 h. Colonies grown on PDA, circular, mycelium flat, dense, reaching 30–40 mm diam. after 30 days of incubation at 25 °C, grey or brown, reverse-side dark brown.

##### Material examined.

Philippines, Iloilo, Calinog, on submerged decaying wood in a freshwater stream, 3 October 2023, James Ariel Nim, CALINOG-004-03-001 (UPVMI 25–0088), living culture UPVMICC 25–0088.

##### GenBank accession numbers.

ITS = PZ203856, LSU = PZ208526, RPB2 = PZ221686, TEF1-α = PZ221696.

##### Notes.

*Distoseptispora septata* was introduced by Ma et al. (2022) from submerged decaying wood in a freshwater stream in China. Both strains, GZCC 22-0078 (holotype) and UPVMICC 25–0088, were collected as saprobes on submerged woody substrates in freshwater habitats and conform well to the generic circumscription of *Distoseptispora*. *Distoseptispora septata* GZCC 22-0078 possesses longer conidiophores (23–86 μm, up to 6-septate) and comparatively shorter conidia (22–179 × 10–16 μm, mean 89 × 13 μm) with up to 25 distosepta and reportedly verrucose conidial walls (Ma et al. 2022). In contrast, UPVMICC 25–0088 is characterised by shorter conidiophores (≤ 20 μm, 2–3-septate) and markedly longer conidia (129–220 × 9–15 μm, mean 159.5 × 12.4 μm) bearing up to 46 distosepta. Phylogenetically, UPVMICC 25–0088 clusters with the holotype strain GZCC 22–0078 with maximal statistical support (100% MLBS, 1.00 BYPP). Nucleotide comparisons reveal no differences in the ITS and RPB2 loci, while minor variation is observed in LSU (3/821 bp; 0.37%) and TEF1-α (2/915 bp; 0.22%). Although differences were observed in conidiophore and conidial dimensions, species delimitation in *Distoseptispora* is based on the integration of morphological characteristics and multilocus phylogenetic evidence and morphological variation amongst conspecific collections has been documented within the genus (Hyde et al. 2020; Yang et al. 2021). Therefore, the strong phylogenetic support and low sequence divergence across multiple loci confirm that UPVMICC 25–0088 and GZCC 22–0078 represent conspecific isolates of *D. septata*, with the observed morphological differences falling within the species’ phenotypic range. This study represents a new geographic record of *D. septata*.

### Synonymisation of *D. thailandica* under *D. phangngaensis*

*Distoseptispora phangngaensis* Jing Yang, Maharachch. & K.D. Hyde, Mycological Progress 17(5): 609 (2018).

=*Distoseptispora thailandica* Tibpromma & K.D. Hyde, Fungal Diversity 93: 79 (2018).

*Distoseptispora phangngaensis* and *D. thailandica* share key diagnostic features of the genus, including macronematous, mononematous conidiophores and acrogenous, distoseptate conidia. However, *D. phangngaensis* is readily distinguishable by its longer, elongate-obclavate conidia (165–350 × 14–19 μm), which are dark olivaceous to brown and conidiophores that taper distally (Yang et al. 2018). In contrast, *D. thailandica* produces comparatively shorter, oblong to cylindrical conidia (130–230 × 13.5–17 μm) and a reddish-brown pigmentation (Tibpromma et al. 2018). Ecologically, both species were described from Thailand; however, *D. phangngaensis* is associated with freshwater habitats, whereas *D. thailandica* occurs terrestrially on decaying leaves of *Pandanus* sp. Phylogenetically, *D. phangngaensis*MFLUCC 16–0857 clusters with the ex-type strain of *D. thailandica*MFLUCC 16–0270 with strong statistical support. Nucleotide comparisons reveal low levels of divergence across the analysed loci, with 5/516 bp (0.97%) differences in ITS, 3/800 bp (0.38%) in LSU and 3/933 bp (0.32%) in TEF1-α. The minimal sequence variation observed, together with robust phylogenetic support, indicates that these taxa represent conspecific lineages. Based on the principle of priority, *D. thailandica* is best treated as a synonym of *D. phangngaensis*. The observed differences in conidial size and pigmentation are considered insufficient to justify species-level delimitation given the minimal sequence variation and strong phylogenetic support. Similar variation in conidial characteristics, septation and pigmentation has been reported amongst members of *Distoseptispora* which indicates that any observed morphological differences are interpreted as ecological plasticity associated with substrate and habitat conditions (Yang et al. 2018; Liao et al. 2025).

### Synonymisation of *D. mengsongensis* under *D. fasciculata*

*Distoseptispora fasciculata* W. Dong, H. Zhang & K.D. Hyde, Mycosphere 12(1): 36 (2021).

= *Distoseptispora mengsongensis* Jing W. Liu, X.G. Zhang & Jian Ma, Journal of Fungi 9(no. 470): 5 (2023).

*Distoseptispora fasciculata* and *D. mengsongensis* share typical generic features, such as macronematous, mononematous conidiophores and acrogenous, distoseptate conidia; however, they differ notably in ecology and micromorphology. *D. fasciculata* is freshwater-inhabiting on submerged decaying wood, forming distinctly fasciculate, shorter conidiophores (12–16 × 5–6 μm) with fewer septa (0–1), whereas *D. mengsongensis* occurs in terrestrial habitats on dead branches and develops longer conidiophores (17–54 × 4.5–7 μm) with greater septation (1–5) (Dong et al. 2021; Liu et al. 2023). Conidia of both species are obclavate to subcylindrical and distoseptate, but *D. fasciculata* produces generally wider conidia (10–16.5 μm) with a characteristic cuneiform base and a faintly pigmented scar, whereas *D. mengsongensis* has narrower conidia (6–13 μm), often showing septal constrictions and occasional percurrent regeneration from the apex. Additionally, *D. fasciculata* conidia tend to be more frequently curved and display a broader septation range (10–40 distosepta) compared to the slightly narrower range (15–31 distosepta) in *D. mengsongensis*. Phylogenetically, *D. fasciculata* KUMCC 19-0081 clusters with the ex-type strain of *D. mengsongensis*HJAUP C2126 with strong statistical support (100% MLBS, 1.00 BYPP). Nucleotide comparisons reveal low to moderate levels of divergence across the analysed loci, with 7/551 bp (1.27%) differences in ITS, 13/582 bp (2.23%) in LSU and no differences in TEF1-α (0/927 bp; 0%). The absence of variation in the protein-coding locus and the overall low sequence divergence, together with robust phylogenetic support, suggest that these taxa represent conspecific lineages. Notably, *D. mengsongensis* has been observed primarily in culture, whereas *D. fasciculata* has been documented from its natural substrate. Based on the principle of priority, *D. mengsongensis* is treated as a synonym of *Distoseptispora fasciculata*. The observed morphological differences are interpreted as ecological plasticity associated with differences in substrate and habitat conditions, consistent with studies (see Jeewon and Hyde (2016); Yang et al. (2021)) demonstrating that morphological characters in fungi may vary amongst collections and environmental conditions.

## Discussion

*Distoseptispora* species are commonly found in terrestrial and freshwater ecosystems. Recent studies have documented their occurrence across various habitats, including decaying plant substrates in forest ecosystems (Hu et al. 2023; Liao et al. 2025), terrestrial palm substrates (Konta et al. 2023), decaying bamboo (Sun et al. 2020; Monkai et al. 2020; Zhai et al. 2022) and wood in freshwater environments (Song et al. 2020; Dong et al. 2021; Shen et al. 2024; Liu et al. 2025; Shen et al. 2025). Liu et al. (2025) suggested that *Distoseptispora* exhibits relatively weak host specificity for leaves and predominantly colonises decaying wood, consistent with the substrates observed in the present study. As saprobes, species of *Distoseptispora* contribute to the decomposition of lignocellulosic substrates and nutrient cycling in both terrestrial and aquatic ecosystems and recent studies have further extended their known ecological range to marine environments, exemplified by *D. mangrovei* (Li et al. 2026).

The rapid taxonomic expansion of *Distoseptispora* is attributed to integrative taxonomic approaches combining morphology with multigene phylogenetic analyses (e.g. ITS, LSU, TEF1-α and RPB2) crucial in delineating members of morphologically similar genera. This underscores the effectiveness of integrative taxonomy in uncovering hidden diversity and demonstrates that *Distoseptispora* remains insufficiently explored in marine habitats. The combined dataset has resolved members of the genus with high bootstrap support values. However, several deeper clades remain weakly supported which suggests that, while the current multigene phylogenetic analyses report effective species-level delimitation, it provides limited support in resolving deeper evolutionary relationships within the genus. One limitation of the present study is the absence of the LSU sequence for *Distoseptispora antiqueana*. Repeated optimisation of LSU amplification was undertaken; however, insufficient quality for sequencing was still observed. *D. antiqueana* is recovered as a distinct lineage, based on the combined ITS, TEF1-α and RPB2 dataset and is further supported by the morphological characteristics observed. Sequencing this locus is suggested in future studies to obtain a complete multilocus dataset for this species. Future studies incorporating broader taxon sampling as well as the addition of other protein-coding loci or whole genome sequences will improve the resolution of deeper relationships within the genus, providing a comprehensive understanding of the evolutionary history of *Distoseptispora*.

Amongst aquatic *Distoseptispora* species, *Distoseptispora iloiloensis*, *D. panayensis* and *D. antiqueana* are introduced as novel species of *Distoseptispora* from the Philippines. *Distoseptispora iloiloensis* and *D. panayensis* were isolated from freshwater environments in the country, while *D. antiqueana* represents a marine taxon. Notably, the first geographical record of *Distoseptispora septata* is documented in this paper. A summary of the morphological characteristics of aquatic *Distoseptispora* species is provided in Table 2. The discovery of novel aquatic *Distoseptispora* species in the Philippines reveals the presence of diverse undocumented fungal taxa in these habitats and highlights the need for further exploration in the country’s aquatic ecosystems.

**Table 2.** Comparative summary of morphological characteristics, habitat preferences, host substrates and geographic distribution of aquatic *Distoseptispora* species.

**Species**	**Conidiophore (μm)**	**Conidia**			**Aquatic habitat**	**Host/ Locality**	**Reference**
		**Size (µm)**	**Septation**	**Morphology**			
*D. amniculi*	90–180 × 3–4.5	85–167 × 9–11.8	(7–)12–24- septate	Obclavate, rostrate, olivaceous brown, greyish-brown or mid-brown, paler towards the apex	Freshwater	Unidentified submerged wood, Thailand	Yang et al. (2021)
*D. appendiculata*	62–86 × 4.5–5.5	67–89 × 10–16	13–17-distoseptate	Obpyriform or obclavate, olivaceous or dark brown, with gelatinous sheath around tip	Freshwater	Unidentified submerged wood, Thailand	Luo et al. (2019)
*D. antiqueana*	12–35 × 7–11	139–268 × 9–16	Up to 39 distoseptate	Obclavate, cylindrical or rostrate, elongated, smooth-walled, straight or slightly curved, guttulate, olivaceous to dark brown	Intertidal	Unidentified submerged wood, Philippines	This study
*D. aqualignicola*	20–50 × 7–10	110–375 × 14–20	16–62 distoseptate	Cylindrical to obclavate, rostrate, rounded apex and subcylindrical to conical-truncate basal cells	Freshwater	Unidentified submerged wood, China	Zhang et al. (2022)
*D. aquamyces*	90–190(–240) × 5–8	41–94(–104) × 10.5–12.5	4–8 euseptate	Obclavate, rostrate, straight or curved, tapering towards the rounded apex, brown at the base, smooth, thin-walled, subhyaline to pale-brown at the apex	Freshwater	Unidentified submerged wood, China	Zhang et al. (2022)
*D. aquatica*	29–41 × 7–9	110–157 × 13.5–16.5	15–28 distoseptate	Obclavate, dark brown with bluish-green to malachite green tinge	Freshwater	Unidentified submerged wood, China	Su et al. (2016)
*D. aquisubtropica*	16–83 × 5–11	43–278 × 11–19	16–31 distoseptate	Obclavate or lanceolate, rostrate, straight or slightly curved, verrucose, guttulate, thick-walled, smooth-walled, pale brown or dark brown, olivaceous, sometimes with conspicuous hyphae attached to the conidium,	Freshwater	Unidentified submerged wood, China	Ma et al. (2022)
*D. arecacearum*	70–140 × 5.1–6.3	25–60 × 7–17	4–10 distoseptate	Cylindrical, obclavate to obpyriform or irregular, brown, sometimes with percurrent regeneration forming a secondary conidium from the conidial apex	Freshwater	Submerged rachis of *Licuala paludosa* sp., Thailand	Karimi et al. (2024)
*D. atroviridis*	100–167 × 2.7–4	31–43 × 13–20	6-septate	Ellipsoidal to obovoid, dark green, subhyaline at the basal cell, smooth-walled, guttulate	Freshwater	On submerged decaying twig, China	Yang et al. (2021)
*D. foveolata*	(55–)69–126(–168) × 10–12	(55–)69–126 (–168) × 10–12	7–18-euseptate	Obclavate, rostrate, olivaceous to pale or dark brown	Freshwater	Unidentified submerged decaying wood, China	Shen et al. (2024)
*D. bambusicola*	64–116 μm × 4–7	72–193 μm × 7.5–14.5	Up to 16 distoseptate	Obclavate or lanceolate, rostrate, straight or slightly curved, guttulate, pale brown	Freshwater	Unidentified submerged decaying wood, China	Jayawardena et al. (2023)
*D. bangkokensis*	37–55 × 3–4	400–568 × 13–16	Multi-distoseptate	Elongate, obclavate, rostrate, dark olivaceous to dark brown	Freshwater	Unidentified submerged wood, Thailand	Shen et al. (2021)
*D. cangshanensis*	44–68 × 4–8	58–166 (–287) × 10–14	Multi-distoseptate	Obclavate or lanceolate, rostrate, olivaceous or brown	Freshwater	Unidentified submerged wood, China	Luo et al. (2018)
*D. chiangraiensis*	35–64 × 4.0–6	68–172 × 6.5–11	15–25 distoseptate	Obclavate, rostrate, truncate at base, rounded at apex, slightly constricted at septa, thick-walled, olivaceous to dark brown, pale brown to subhyaline towards the apex	Freshwater	On submerged decaying bamboo culms, Thailand	Manawasinghe et al. (2024)
*D. chinensis*	16.5–44 × 5.5–9	81–283 × 10–19	Up to 40 distoseptate	Obclavate or lanceolate, rostrate, straight or slightly curved, olivaceous to dark brown	Freshwater	Submerged decaying wood, China	Hyde et al. (2021)
*D. clematidis*	(8–)15–25 μm × 5–8 μm	(90–)126–245(–303) × 14–20	(17–)20–41(–49)distoseptate	Obclavate, elongated, straight or curved, truncate at the base, rounded at the apex, smooth, thick-walled, brown with a green tinge, sometimes with percurrent proliferation	Freshwater	Unidentified submerged wood, China	Zhang et al. (2022)
*D. crassispora*	14–27 × 6–10	95–197(–214) × 13–24	15–36(–41) distoseptate	obclavate, rostrate, mostly curved, truncate at the base, rounded at the apex, smooth, thick-walled, brown with a green tinge	Freshwater	Unidentified submerged wood, China	Zhang et al. (2022)
*D. curvularia*	11–28 × 5–9	(60–)100–200(–314) × 12–19	(9–)16–48(–59)-distoseptate	Obclavate, rarely oblong, rostrate, mostly curved, truncate at the base, rounded at the apex, smooth, thick-walled, brown with a green tinge	Freshwater	Unidentified submerged wood, China	Zhang et al. (2022)
*D. cylindricospora*	105–157 × 6.5–8.5	136.5–278 × 8.5–11	20–65 distoseptate	Cylindrical to elongated, straight or slightly curved, truncated at the base, rounded at the apex, greenish-brown to dark brown	Freshwater	Submerged decaying wood, China	Phukhamsakda et al. (2022)
*D. davidii*	64–104 × 5–8	93–177 × 12–17	Multi-distoseptate	Obclavate, rostrate, straight or curved, olivaceous brown	Freshwater	On submerged rotting wood, China	Xiao et al. (2025)
*D. dehongensis*	45–80 × 4–5	17–30 × 7.5–10	3–5 distoseptate	Obpyriform to obclavate, broad cylindrical or irregular, olivaceous	Freshwater	Unidentified submerged wood, China	Hyde et al. (2019)
*D. dujuanhuensis*	(119–)141–175(–191) × 5–7(–8)	(33–)49–89(–109) × (7–)9–12	7–9 distoseptate	Obclavate, rostrate, truncate at base, tapering towards the apex, straight or slightly curved, pale brown	Freshwater	On submerged decaying wood, China	Shen et al. (2025)
*D. effusa*	72–171 × 5–6.5	35.5–113 × 7–12.5	4–9 distoseptate	Obclavate,rostrate, smooth-walled, olivaceous brown to dark brown, sometimes slightly paler at the apex, straight or slightly curved,	Freshwater	Submerged decaying wood, China	Yang et al. (2021)
*D. eleiodoxae*	71–161 × 5–6.5	31.5–48 × 13.5–15.8	6–7 euseptate	Obpyriform, rostrate, verrucose, brown with dark brown to black cells in the middle, paler towards the apex	Freshwater	Submerged rachis of *Eleiodoxa conferta*, Thailand	Karimi et al. (2024)
*D. euseptata*	19–28 × 4–5	37–54 × 8–9	4–7 euseptate	Obpyriform to obclavate, often constricted at septa, olivaceous	Freshwater	Unidentified submerged wood, China	Li et al. (2021)
*D. fasciculata*	12–16 × 5–6	46–200 × 10–16.5	10–40 distoseptate	Subcylindrical to obclavate, olivaceous when young, dark brown when mature	Freshwater	Unidentified submerged wood, Thailand	Dong et al. (2021)
*D. fluminicola*	21–33 × 5.5–6.5	125–250 × 13–15	17–34 distoseptate	Oblong, obclavate, cylindrical or Rostrate, brown with green tinge	Freshwater	Unidentified Submerged wood, China	Su et al. (2016)
*D. fusiformis*	40–110 × 3.5–5.8	35–52 × 13.5–22	6–8-septate	Ellipsoidal to fusiform, dark olivaceous brown to dark brown, pale brown at both ends,	Freshwater	Unidentified Submerged wood, China	Yang et al. (2021)
*D. guanxiensis*	79.5–141 × 3.2–5.5 µm	36–122 × 7.5–15 µm	7–15 distoseptate	Obclavate or obspathulate, rostrate, tapering towards the apex, straight or slightly curved, olivaceous to brown, becoming pale brown to subhyaline towards the apex	Freshwater	Unidentified submerged wood, China	Tian et al. (2026)
*D. guttulata*	55–90(–145) × 3.5–5.5	75–130(–165) × 7–11	11–14(–20) euseptate	Obclavate or lanceolate, rostrate, mid- to dark brown or olivaceous	Freshwater	Unidentified submerged wood, Thailand	Yang et al. (2018)
*D. hongheensis*	(96–)120–176(–206) × 5–7.5	(53–)80–117(–141) × 8–10	11–22 distoseptate	Obclavate, arrow lanceolate, rostrate, straight or slightly curved, guttulate, yellowish-brown to brown with bluish-green tinge	Freshwater	On submerged decaying wood, China	Shen et al. (2025)
*D. iloiloensis*	14–34 × 4–8 μm	(82–)102–200 (–385) × 6–19	Up to 39 distoseptate	Obclavate, cylindrical or rostrate, olivaceous to dark brown	Freshwater	Unidentified submerged wood, Philippines	This study
*D. jingdongensis*	(31–)44–59(–67) × 4–6	(78–)88–120(–146) × (8–)11–14(–18)	14–20 distoseptate	Obclavate, cylindrical, rostrate, truncate at base, tapering towards the rounded apex, straight or slightly curved, pale brown with olivaceous tinge	Freshwater	On submerged decaying wood, China	Shen et al. (2025)
*D. keviniana*	55–120 × 4–8	41–103 × 10–15	Up to 12 distoseptate	Obclavate, rostrate, straight or slightly curved, olivaceous brown	Freshwater	On decaying wood	Xiao et al. (2025)
*D. lancangjiangensis*	144–204 × 5–6	64–84 × 9–10	3–10 euseptate	Narrowly obclavate or obspathulate, tracted at base, tapering towards apex, brown to dark brown	Freshwater	Unidentified submerged wood, China	Shen et al. (2021)
*D. lanceolatispora*	120–190 × 4–8	31–90 × 9.5–15	5–13 distoseptate	Fusiform or lanceolate, rostrate, olivaceous to olivaceous brown, verrucous, with or without apical, hyaline appendages	Freshwater	Unidentified submerged wood, China	Chen et al. (2024)
*D. lignicola*	84–124 × 4–5	60–108 × 7–9	5–9 euseptate	Obclavate, curved, brown	Freshwater	Unidentified submerged wood, Thailand	Luo et al. (2019)
*D. longiconidiophora*	196–409 (–486) × 4.5–6.5 μm	100–142 × 5.5–8 μm	13–27 distoseptate	Cylindrical to obclavate, straight or slightly curved, truncate at the base, tapering towards the apex, olivaceous brown to dark brown	Freshwater	Unidentified submerged wood, China	Tian et al. (2026)
*D. longissima*	204–332 × 5–8.5	45–167 × 6.5–10	6-24 euseptate	Obclavate, rostrate, curved, rounded at the apex, truncate at the base, tapering towards apex, smooth, thin-walled, pale green to pale brown	Wetland	On a submerged decaying wood, China	Liu et al. (2025)
*D. longispora*	17–37 × 6–10	189–297 × 16–23	31–56 distoseptate	Obclavate, elongated, brown to yellowish-brown	Freshwater	Unidentified submerged wood, China	Song et al. (2020)
*D. mangrovei*	8–37 × 4–8	12–122 × 6–13 μm	7–22 distoseptate	Obclavate, elongated, subhyaline when young, olivaceous or brown when mature	Mangrove	Decaying wood of *Sonneratia apetala*	Li et al. (2026)
*D. meilingensis*	69–192 × 4–7	32–64.5 × (7–) 9–12.5	5–7-distoseptate	Obclavate, mostly bright brown when mature	Freshwater	Dead bamboo culms, China	Zhai et al. (2022)
*D. monospora*	62–117 × 3–5.1	27–126 × 6.2–8.9	2–13 euseptate	Narrowly obclavate, rostrate, tapering towards the apex, straight or slightly curved at the base, smooth to slightly verrucose walled, brown	Freshwater	On submerged decaying wood, China	Hongsanan et al. (2025)
*D. motuoensis*	85–174 × 6–9	39–88 × 9–15	7–12 distoseptate	Obclavate, thick-walled, straight or slightly flexuous, pale brown or olive-green	Freshwater	On submerged decaying wood, China	Li et al. (2024)
*D. multiseptata*	29–47 × 4–6	147–185 × 12–14	Multi-distoseptate	Obclavate, rostrate, dark olivaceous green	Freshwater	Unidentified submerged wood, Thailand	Hyde et al. (2016b)
*D. neorostrata*	93–117 × 5.5–6.5	109–147 × 13–15	Multi-distoseptate	Obclavate, rostrate, dark olivaceous to mid- or dark brown	Freshwater	Unidentified submerged wood, Thailand	Luo et al. (2019)
*D. nonrostrata*	105–160 × 4.5–7	22–51 × 8–14	4–10 distoseptate	Oblong, obclavate or narrowly obpyriform, mostly non-rostrate, rarely rostrate, straight or curved, truncate at the base, smooth, thick-walled, pale olivaceous or pale brown	Freshwater	Unidentified submerged wood, China	Zhang et al. (2022)
*D. obclavata*	117.5–162.5 × 5–7	46–66 × 9–11	9-11 distoseptate	Obclavate, olivaceous to pale or dark brown, guttulate	Freshwater	Unidentified submerged wood, Thailand	Luo et al. (2019)
*D. obpyriformis*	97–119 × 5–7	53–71 × 12–16	9–11 distoseptate	Obpyriform, olivaceous to pale or dark brown, guttulate	Freshwater	Unidentified submerged wood, China	Luo et al. (2018)
*D. pachyconidia*	11–27 × 4–9	42–136 × 14–22	8–21 distoseptate	Obclavate, lanceolate, rostrate or not, straight or curved, truncate at the base, tapering towards the rounded apex, smooth, thick-walled, pale brown with a green tinge	Freshwater	Unidentified submerged wood, China	Zhang et al. (2022)
*D. panayensis*	66–134 × 4–8	82–174 × 7–12	13–23-distoseptate	Obclavate, rostrate, elongated, olivaceous brown to greyish-brown	Freshwater	Unidentified submerged wood, Philippines	This study
*D. phangngaensis*	18–30(–40) × 4.3–6.5	165–350 × 14–19	Multi-distoseptate	Elongate, obclavate, rostrate, dark olivaceous to mid- or dark brown	Freshwater	Unidentified submerged wood, Thailand	Yang et al. (2018)
*D. pseudoaquisubtropica*	31–50 × 7.5–10	284–423 × 12–19	Multi-distoseptate	Long-obclavate, rarely branched, with truncate base, greenish-brown	Freshwater	On decaying wood, China	Xiao et al. 2025
*D. polyblasta*	Up to 310 × 3–6	47–85 × 6–10.5	6–9-euseptate	Obclavate, with truncate base, olivaceous brown to brown	Freshwater	On decaying bamboo culms, Thailand	Xiao et al. 2025
*D. pulchra*	(45–)63–86(–107) × 4.5–6.6	(87–)92–127(–154) × 11–14	19–26(–36) distoseptate	Obclavate, rostrate, truncate at base, tapering towards the rounded apex, straight or slightly curved, brown with olivaceous tinge	Freshwater	On submerged decaying wood, China	Shen et al. (2025)
*D. quinqueseptata*	173–311 × 5.8–9	11–39.5 × 4.5–8	2–5 distoseptate	Subcylindrical or obclavate, straight or curved, olivaceous to brown, apex rounded	Wetland	On a submerged decaying wood, China	Liu et al. (2025)
*D. rayongensis*	75–125 × 3.5–5.5	(36–)60–106(–120) × 9–14.5	9–13-euseptate, rarely 14–15-septate	Obclavate or obspathulate, rostrate, pale brown or pale olivaceous, with percurrent proliferation	Freshwater	Unidentified submerged wood, Thailand	Hyde et al. (2020)
*D. rostrata*	82–126 × 5–7	115–155 × 9–11	(15–)18–23 distoseptate	Obclavate or lanceolate, rostrate, olivaceous to pale brown	Freshwater	Unidentified submerged wood, China	Luo et al. (2018)
*D. saprophytica*	50–140 × 3.2–4.2	14.5–30 × 4.5–7.5	2–6 distoseptate	Subcylindrical to obclavate, olivaceous to brown	Freshwater	Unidentified submerged wood, Thailand	Dong et al. (2021)
*D. septata*	Up to 20	129–220 × 9–15	Up to 46- distoseptate	Obclavate, cylindrical or rostrate, elongated, straight or slightly curved, guttulate, olivaceous to dark brown	Freshwater	Unidentified submerged wood, Philippines	This study
*D. septata*	23–86 × 3–7	22–179 × 10–16	Multi-distoseptate and up to 25-septate	Obclavate, rostrate, straight or slightly curved, guttulate, pale brown or dark brown, olivaceous-green	Freshwater	Unidentified submerged wood, China	Ma et al. (2022)
*D. solitaria*	91–188 × 5–9	32–54 × 8–11	Mostly 9-distoseptate	Obclavate, straight or slightly curved, olivaceous to dark brown	Freshwater	On decaying wood, China	Xiao et al. (2025)
*D. songkhlaensis*	70–90 × 4–5.5	44–125 × 9–14.5	9–16 distoseptate	Obclavate, constricted at septa, olivaceous to brown	Freshwater	Unidentified submerged wood, Thailand	Dong et al. (2021)
*D. submersa*	55–73 × 7–9	95–123 × 15–19	17–23(–28) distoseptate	Obclavate, brown to dark brown or olivaceous	Freshwater	Unidentified submerged wood, China	Luo et al. (2018)
*D. suoluoensis*	80–250 × 4.5–5.8	(65–)80–125(–145) × 8–13	8–10 euseptate	Narrowly obclavate or obspathulate, yellowish-brown or dark olivaceous, verrucose, with percurrent proliferation	Freshwater	Unidentified submerged wood, China	Yang et al. (2018)
*D. suae*	(21–)25–41(–53) × 4–5	(77–)81–101(–109) × 8–10	3–12 euseptate	Obclavate to rostrate, bent at the second or third cell at the base, brown to dark brown, guttulate, verrucose	Freshwater	On submerged decaying branches, China	Shen et al. (2024)
*D. tectonae*	19.5–95 × 4.5–9	45–270 × 11–16	10–40- distoseptate	Obclavate, brown to dark brown or olivaceous	Freshwater	Dead twig of *Tectona grandis* Thailand	Hyde et al. (2016b)
*D. tongrensis*	29–54 × 4.2–5.2	23–37 × 6.5–10.2	Mostly 5–9 distoseptate	Obclavate to clavate sometimes fusiformis, straight or slightly curved, truncate at the base	Wetland	On a submerged decaying wood, China	Liu et al. (2025)
*D. thysanolaenae*	41–59 × 4–5	46–87 × 9–12	6–19-septate	Obclavate, elongated, straight or slightly curved, dark greyish-brown to light yellow green	Freshwater	On submerged decaying wood, China	Shen et al. (2021)
*D. velvetica*	75–107 × 4–8	46–86 × 10–25	Multi-distoseptate	Obclavate, rostrate, straight or slightly curved, olivaceous brown	Freshwater	On submerged decaying wood, China	Xiao et al. (2025)
*D. verrucosa*	92–250 × 4.7–6.3	41–63 × 8.8–12.6	6–8 septate	Obclavate, rostrate, upper part tapering towards the apex, olivaceous brown, becoming paler at the apex, verrucose, guttulate	Freshwater	On submerged decaying wood, China	Yang et al. (2021)
*D. wuzhishanensis*	16–56 × 5–7	76–143 × 11–17	up to 22 distoseptate	Obclavate, rostrate, straight, or slightly curved, verrucose, guttulate, thick-walled, pale brown or dark brown, olivaceous-green and yellow	Freshwater	Unidentified submerged wood, China	Ma et al. (2022)
*D. xinpingensis*	(97–)105–149(–175) × 4–5	(95–)107–139 (–155) × (7–)8–9(–10)	8–12 euseptate	Obclavate, brown, sometimes a second conidium proliferates at the top of the conidia,	Freshwater	On submerged decaying branch, China	Shen et al. (2024)
*D. xishuangbannaensis*	19–52 × 5–7	88–269 × 11–15	Up to 42 distoseptate	Cylindrical-obclavate, rostrate, straight, or slightly curved, guttulate, thick-walled, smooth-walled, green-brown or brown	Freshwater	On submerged decaying wood, China	Ma et al. (2022)
*D. yunnanensis*	131–175 × 6–7	58–108 × 8–10	6–10 euseptate	Obclavate, rostrate, mid-olivaceous to brown	Freshwater	Unidentified submerged wood, China	Li et al. (2021)
*D. yongxiuensis*	112–253 × 4–9	46–74 (–86) × 10–13 (–16)	6–9 euseptate	Obclavate or obspathulate, olivaceous to yellowish-brown or brown, guttulate	Freshwater	Dead bamboo culms, China	Zhai et al. (2022)
*D. yunjushanensis*	100–175 × 5.5–10	39–67.5 (–77) × (7–)9.5–13.5 (–16.5)	7–13 distoseptate	Obpyriform or obclavate, olivaceous when young, dark brown when mature	Freshwater	Dead bamboo culms, China	Zhai et al. (2022)

## Supplementary Material

XML Treatment for Distoseptispora antiqueana

XML Treatment for Distoseptispora iloiloensis

XML Treatment for Distoseptispora panayensis

XML Treatment for Distoseptispora septata

## Additional information

### Artificial Intelligence (AI) use

The authors accept full responsibility for the content of the manuscript, including the disclosure of any use of AI.

No AI tools were used in the preparation of this manuscript.

### Author ORCIDs

J.F.E. Bagacay https://orcid.org/0009-0009-4717-217X

G.M. Candole https://orcid.org/0009-0005-1662-7099

J.C. Velo https://orcid.org/0009-0009-6243-1440

F.R. Sara https://orcid.org/0009-0003-6099-9741

M.A. Siccio https://orcid.org/0009-0006-0986-3226

L.A. Opiña https://orcid.org/0009-0003-8648-1510

E.B.G. Jones https://orcid.org/0000-0002-7286-5471

M.S. Calabon https://orcid.org/0000-0002-6603-1876

### Data availability

All of the data that support the findings of this study are available in the main text.
